# Prehospital Identification of Large Vessel Occlusions Using Modified National Institutes of Health Stroke Scale: A Pilot Study

**DOI:** 10.3389/fneur.2021.643356

**Published:** 2021-05-14

**Authors:** William D. Mulkerin, Ilanit Spokoyny, Jonathan T. Francisco, Brandon Lima, Megan D. Corry, Matthew J. R. Nudelman, Kian Niknam, Ian P. Brown, Michael A. Kohn, Prasanthi Govindarajan

**Affiliations:** ^1^Department of Emergency Medicine, Stanford University School of Medicine, Palo Alto, CA, United States; ^2^Department of Neurology, California Pacific Medical Center, San Francisco, CA, United States; ^3^Emergency Medical Services Division of Woodside Fire Protection District, Portola Valley, CA, United States; ^4^City College of San Francisco, San Francisco, CA, United States; ^5^School of Medicine, University of California, San Francisco, San Francisco, CA, United States

**Keywords:** stroke—diagnosis, stroke scale, paramedic, ambulance, emergency care, prehospital/EMS

## Abstract

Stroke identification is a key step in acute ischemic stroke management. Our objectives were to prospectively examine the agreement between prehospital and hospital Modified National Institutes of Health Stroke Scale (mNIHSS) assessments as well as assess the prehospital performance characteristics of the mNIHSS for identification of large vessel occlusion strokes.

**Method:** In this prospective cohort study conducted over a 20-month period (11/2016–6/2018), we trained 40 prehospital providers (paramedics) in Emergency Neurological Life Support (ENLS) curriculum and in mNIHSS. English-speaking patients aged 18 and above transported for an acute neurological deficit were included. Using unique identifiers, we linked the prehospital assessment records to the hospital record. We calculated the agreement between prehospital and hospital mNIHSS scores using the Bland-Altman analysis and the sensitivity and specificity of the prehospital mNIHSS.

**Results:** Of the 31 patients, the mean difference (prehospital mNIHSS—hospital mNIHSS) was 2.4, 95% limits of agreement (−5.2 to 10.0); 10 patients (32%) met our a priori imaging definition of large vessel occlusion and the sensitivity of mNIHSS ≥ 8 was 6/10 or 0.60 (95% CI: 0.26–0.88) and the specificity was 13/21 or 0.62 (95% CI: 0.38–0.82), respectively.

**Conclusions:** We were able to train prehospital providers to use the prehospital mNIHSS. Prehospital and hospital mNIHSS had a reasonable level of agreement and and the scale was able to predict large vessel occlusions with moderate sensitivity.

## Introduction

Stroke affects 800,000 people in the US annually (1) and treatment is time-dependent. Acute ischemic stroke patients, if eligible, are treated in emergency departments with thrombolytics such as intravenous alteplase within 0–4.5 h from symptom onset (2). Strokes due to large vessel occlusions are treated in the hospitals using mechanical thrombectomy within 24 h from symptom onset when imaging shows a salvageable penumbra or when there is a mismatch between clinical deficit and infarct size (3–6). Although there has been a steady increase in intravenous alteplase use and endovascular thrombectomy (7), missed opportunities still exist (8, 9). Therefore, a global priority is to increase access to these time-sensitive treatments through streamlined care in the prehospital and emergency department settings.

Prehospital providers are the first point of medical contact and provide emergency care to thousands of stroke patients (10) every year. Stroke detection, triage, and transport to stroke centers are critical time-sensitive roles managed by prehospital providers. Despite the significance of the disease, stroke education for prehospital providers is not standardized and remains inconsistent across emergency medical service agencies (11, 12). To achieve greater stroke knowledge and better competencies in stroke detection, a standardized curriculum and ongoing education must occur, and its effects must be assessed. The National Highway Traffic and Safety Administration (NHTSA) in the United States has set forth education of paramedics as one of the priority areas and the objectives of their education agenda include development of a national curriculum, collaboration between academia and prehospital programs, and recognition of prehospital provider education as an academic achievement (13). To implement these objectives for stroke, one of our investigators (MC) partnered with stroke neurologists to create the ENLS (Emergency Neurological Life Support) curriculum for prehospital care, a standardized curriculum on stroke for prehospital providers. Prehospital ENLS provides evidence-based protocols that are designed to address the 1st hour of an acute neurological event. These protocols offer a multidisciplinary approach that promotes interprofessional training and collaborative practice between prehospital EMS providers, nurses, and physicians (14).

In addition to the ENLS, we offered stroke scale training to all of the prehospital providers. A couple of decades back, NIHSS (National Institutes of Health Stroke Scale) was developed to quantify the severity of strokes, and was primarily used in the hospital setting for clinical and research use. Subsequently, prehospital stroke scales were developed using the NIHSS elements for detection of strokes and large vessel occlusions. These scales were simple and designed as a quick tool for use by paramedics and other first responders. However, our prehospital providers articulated a strong preference to use a hospital-based stroke scale in the prehospital setting. They articulated this during the focus group sessions that were conducted to learn about barriers to stroke identification in the prehospital setting. Prehospital providers mentioned that using a hospital-based stroke scale with similar exam elements would enhance communication with hospital providers and facilitate feedback on their exam from the hospital staff (physicians, nurses, and advanced practitioners) (8), analogous to the 12 lead Electrocardiogram [ECG] used to detect Myocardial Infarction in the prehospital and hospital settings.

Although the NIHSS is the clinical standard for stroke evaluations, and used widely for stroke evaluations, prognostication, and clinical trial enrollments, some of the NIHSS items (level of consciousness, ataxia, facial palsy, and dysarthria) are known to be less reliable. The mNIHSS was developed to eliminate the redundant and the less reliable NIHSS elements. It's a 11-item, 0-31 point motor stroke deficit scale with very good reliability and validity and has the sample picture, list of words, and sample sentences from the original NIHSS (15). The mNIHSS is derived from the NIHSS, is comprehensive, more reliable than NIHSS and can be administered in 5–8 min. These features fit our study needs and therefore, we chose to study the performance of the mNIHSS in the prehospital setting.

Our two study objectives were: (1) to assess the feasibility of implementing the prehospital ENLS training curriculum and modified National Institutes of Health Stroke Scale (mNIHSS) skills training, and (2) to examine the sensitivity and specificity of prehospital Modified NIHSS (mNIHSS) for detection of large vessel occlusions in patients with acute neurological deficits. Since NIHSS certification offered by American Heart/Stroke Association (AHA/ASA) is a widely accepted program that promotes mastery of the entire NIHSS and the stroke scale elements, our paramedics were required to complete this standardized training along with the focused mNIHSS skills training.

## Methods

This study was approved by our Institutional Review Board and consent process was waived due to the time-sensitive nature of the disease that was being studied.

In this prospective observational study, all of the prehospital providers served with the Woodside Fire Protection District—Emergency Medical Services Agency. All of the prehospital providers were paramedics and there was no difference in their education level. Prior to patient enrollment, the paramedics completed the three-part training conducted by our investigators (WDM, IS). The training program had the following components: (1) In-person ENLS training by a vascular neurologist (2 h). During this session, the vascular neurologist (IS) reviewed stroke pathophysiology and common presentations, as well as detailed explanation of the NIH Stroke Scale and the modified NIH Stroke Scale components, (2) NIH Stroke Scale certification course offered by the American Heart/Stroke Association program (2 h), and (3) Hands-on training with paramedics modeling mNIHSS assessment to group with feedback by vascular neurologist (2 h).

The training was performed on-site by an emergency physician and a vascular neurologist (WDM, IS). Although these sessions were performed on-site, the content can be easily made available in a telemedicine platform such as Zoom or in a recorded format for a larger audience. During the 20-month period, we did not offer any refresher sessions to the paramedics.

Woodside Fire staffed their ambulance with two firefighter-paramedics who screened and prospectively enrolled patients during the study period 11/2016 through 06/2018. Since this was a feasibility study, a sample size was not estimated. We set an arbitrary cut-off of 20-months based on resource availability. The selection criteria for enrollment were patients 18 years of age or above, had an acute neurological deficit, and were clinically stable for evaluation on-scene. We excluded non-English speaking patients, patients with cognitive or decision-making impairments or classified as vulnerable subjects. Our transport protocol did not change during the study period and we did not exclude any patients with contra-indications to thrombolysis and/or thrombectomy treatment. Prehospital providers collected data on last-seen-normal time, baseline functional status of the patient, medications, and contra-indications for intravenous alteplase. In addition, prehospital providers performed stroke assessments using the Cincinnati Stroke Scale and the prehospital mNIHSS (see Supplementary Material for the subject enrollment form).

After on-scene evaluation, prehospital providers transported patients with suspected stroke to the three destination stroke centers within San Mateo County in the state of California. At the destination hospitals, patients received a standard evaluation using NIHSS evaluation by the stroke neurology team in the emergency departments followed by stroke imaging such as computerized tomography (CT) scan, CT angiogram or magnetic resonance angiogram during the initial evaluation in the emergency department. One of the investigators, a stroke neurologist (IL), abstracted the mNIHSS elements from the stroke team NIHSS to create the hospital mNIHSS, and collected the imaging reports from electronic medical records. Then, using the imaging report, the stroke neurologist (IL) identified large vessel occlusions using the following criteria: occlusion of the intracranial internal carotid artery, the middle cerebral artery M1 or M2 segments, the vertebral artery, the basilar artery, the posterior cerebral artery P1 segment and the anterior cerebral artery A1 segment. For patients without evidence of large vessel occlusions, one of the study investigators (WDM) collected the final discharge diagnosis from the electronic medical records. We used the primary diagnosis documented in the emergency department record if patients were discharged from emergency department and final hospital diagnosis if they were admitted to the hospital. Subsequently, we compiled clinical, imaging and discharge data for each patient and then linked the prehospital and the hospital databases at the patient level using unique identifiers. The investigators did not have access to the prehospital data prior to linking the data.

We assessed the performance characteristics of the prehospital mNIHSS of ≥8 for detection of large vessel occlusion using the imaging diagnosis of large vessel occlusion. To our knowledge, there is no recommended cut-off for the detection of large vessel occlusion and so, we chose a priori cut-off of mNIHSS 8 for an estimated sensitivity of 85% (16). Sensitivity and specificity of mNIHSS at a cutoff of ≥8 were calculated with exact 95% confidence intervals. We reported non-parametric data as median and interquartile range (IQR); Wilcoxon signed-rank test was used to compare prehospital mNIHSS and hospital mNIHSS scores and created a Bland–Altman plot with mean difference and 95% limits of agreement.

## Results

A total of 31 patients met the study criteria. We trained 40 paramedics and patients were enrolled prospectively by 17 paramedics who were assigned to the ambulance transport during the study period. Each patient received an initial stroke assessment and a mNIHSS by the prehospital provider during the ambulance transport and an assessment by the stroke neurologist in the emergency department. The average (SD) time-difference between the initiation of prehospital transport and emergency department arrival was 15.5 min (SD ± 4.7). The patients were transported to three different hospitals in the county. The demographics of the patients were as follows: mean age was 62 years (SD ± 12), male (15, 48%), Non-Hispanic Whites (19, 61%), African-American (1, 0.03%), Asian (9, 29%), Hispanics (none), and Others (2, 0.06%). Among the 31 suspected stroke patients, 16 (52%) had a stroke diagnosis, 3 (10%) had a transient ischemic attack diagnosis, 8 (26%) had a diagnosis of seizure or sepsis, and 4 had other diagnoses (Delirium, Hypoxia, Hypotension, Progressive supranuclear palsy); 11/16 (69%) had a diagnosis of ischemic stroke and of those, 10/11 (91%) met our a priori imaging definition of large vessel occlusion (Table 1).

**Table 1:**
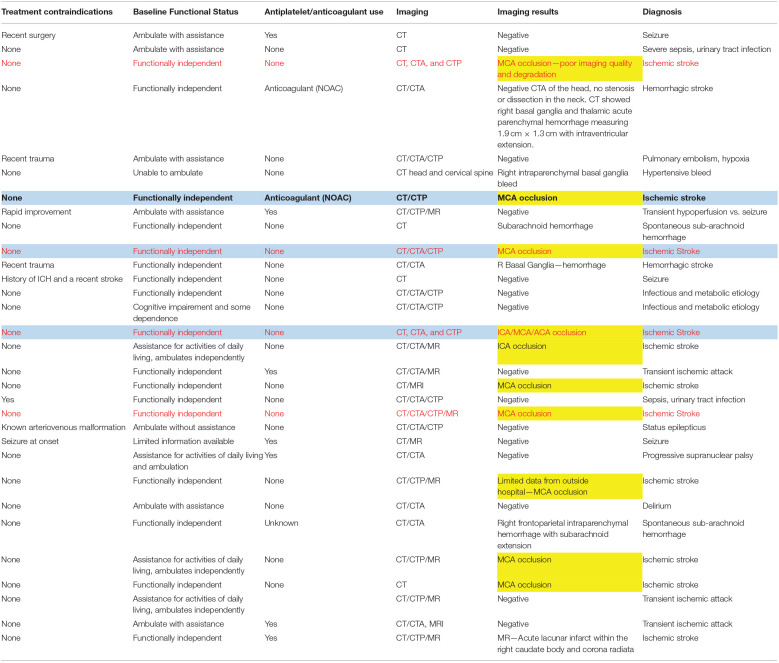
Medical history, imaging, and discharge diagnosis of suspected stroke patients transported by emergency medical services.

*Yellow highlighted row, Large vessel occlusion; Red font, Received thrombolysis; Blue highlighted rows, Underwent Endovascular thrombectomy; CT, computer tomography; CTA, CT angiogram; CTP, CT perfusion; MR, magnetic resonance*.

**Figure 1:**
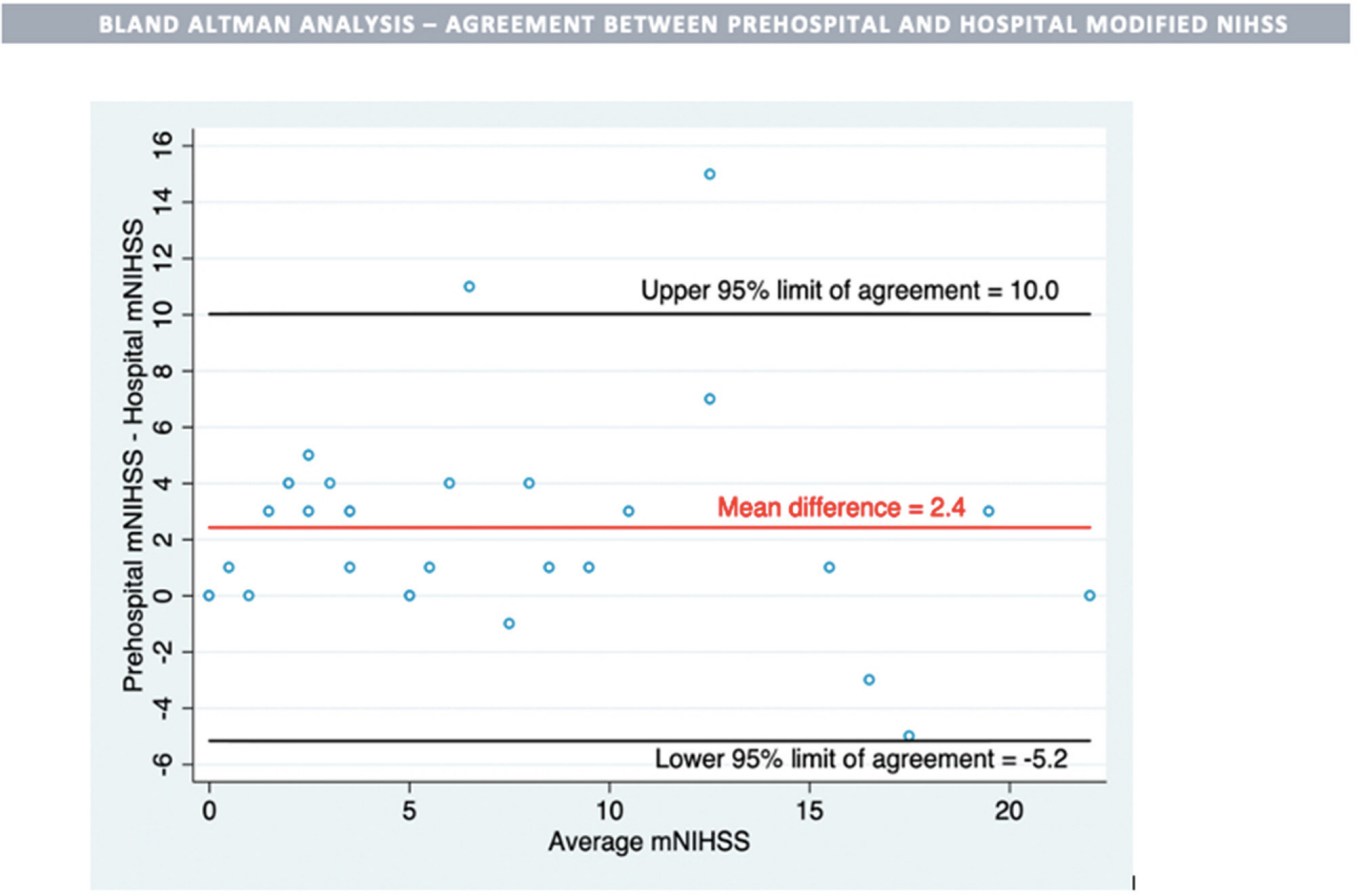
One patient was excluded from comparison due to change in clinical condition during prehospital transport.

The median (IQR) prehospital mNIHSS was higher than the hospital mNIHSS, 6 (4, 15) vs. 4 (1, 9) (*p* < 0.001) for all transported patients. The prehospital score was higher than the hospital score in 22, equal in 6, and lower in three patients. In Bland–Altman analysis, the mean difference (pre-hospital mNIHSS—hospital mNIHSS) was 2.4, 95% limits of agreement (−5.2 to 10.0; Figure 1). Of note, in this cohort, five patients had more than a 4-point difference between the prehospital score and the hospital score.

A total of 14 (45%) patients had a prehospital mNIHSS of ≥8 and 10 (32%) of patients had a hospital mNIHSS of ≥8 indicating a clinical diagnosis of large vessel occlusion. Using the imaging definition described in the Methods section, 10 patients were classified as large vessel occlusions. Based on these numbers, the sensitivity and specificity of the prehospital mNIHSS ≥ 8 were 6/10 or 0.60 (95% CI: 0.26–0.88) and 13/21 or 0.62 (95% CI: 0.38–0.82), respectively. The sensitivity and specificity of a hospital mNIHSS ≥ 8 was 4/10 or 0.40 (95% CI: 0.12–0.74) and 15/21 or 0.71 (95% CI: 0.48–0.89), respectively (Table 2).

**Table 2:** Performance assessment of pre-hospital and hospital modified National Institutes of Health Stroke Scale (mNIHSS) at predicting large vessel occlusions.

		**Pre-hospital mNIHSS**			**Hospital mNIHSS**		
**Diagnosis**	**Total**	**≥8**	**<8**	**Sensitivity (95% CI)**	**≥8**	**<8**	**Sensitivity (95% CI)**
**Large vessel occlusion**	10	6	4	60% (26–88%)	4	6	40% (12–74%)
**Non-large vessel occlusion**	21	8	13	62% (38–82%)	6	15	71% (48–89%)
TIA	3	0	3		0	3	
Seizure or Sepsis	8	4	4		3	5	
Small Vessel Stroke	1	0	1		0	1	
Other	4	2	2		0	4	
Intracranial Hemorrhage	5	2	3		3	2	

## Discussion

This is the first US study that we are aware of to examine the feasibility of a hospital-based stroke scale use in the prehospital setting.

Our major finding was that the prehospital stroke exam using mNIHSS was quite reliable although the levels of agreement varied widely. Similar findings were reported by the authors of a national study that examined the reliability of the NIHSS among the subjects who took the NIHSS certification offered by the National Stroke Association (17). The study demonstrated that a 4-point difference was common among those rating the video vignettes. Even after removing 2 of the less reliable items, the variance persisted. In a study conducted in Norway, after a rigorous 1-day training followed by NIHSS certification, trained anesthesiologists performed a prehospital NIHSS and this was compared with the hospital NIHSS performed by a neurologist. In their study of 40 patients, they reported a mean difference of 0.85, a much better agreement among their prehospital and hospital providers (18).

Our second finding was that mNIHSS had moderate sensitivity for the large vessel occlusion. A prehospital study of NIHSS-8 scale reported a higher sensitivity for large vessel occlusion when compared to our study. However, the NIIHSS-8 scale elements were different from the mNIHSS and NIHSS-8 included facial palsy but did not use sensation and aphasia elements of the scale (19). Also, this scale was applied to a subset of the suspected stroke patients and the hospital physicians applied the scale within 5 min of the stroke exam by prehospital providers. The study sample and the timing of the stroke scale may explain better sensitivity between the scales. Another European study assessed the feasibility of a shortened NIHSS for prehospital providers and reported a sensitivity of 70% using a cut-off score of 6 (20). In another study, investigators examined the sensitivity between helicopter emergency service providers and physicians and reported slightly lower sensitivity (56%) than our study for detection of large vessel occlusion when using NIHSS ≥ 12. Similar to our findings, this study reported that prehospital providers were able to use NIHSS and achieve moderate agreement (Kappa = 0.62) with the hospital providers (21). Similar to the sensitivity, the mNIHSS had a moderate specificity for the detection of large vessel occlusions. A recent systematic review on the stroke scale accuracy for diagnosing large vessel occlusions in individuals with suspected stroke reported similar findings. The review described that most of the scales used for prediction of large vessel occlusions were derived from NIHSS, and that only four of the 36 studies reported prehospital application of the stroke scale (22). The sensitivity of the Cincinnati Prehospital Stroke Severity Scale was similar to mNIHSS (58 vs. 60%) but the specificity was higher (77 vs. 62%). The NIHSS sensitivity and specificity in the prehospital setting had a wide range (Sensitivity [32–74%)], Specificity [62–86%]). Prehospital application of the Los Angeles Motor Scale had a sensitivity of 47–62% and a specificity of 70–90%. RACE scale had a prehospital sensitivity of 56% and a specificity of 87%. The observed differences among the different prehospital studies are due to the sampling differences, provider training and skill differences, differences in data collection, and the nature of the study design. Although our study is prospective, our objective was not to compare existing stroke scales and therefore, we did report superiority of mNIHSS over other scales or vice-versa.

Our findings suggest that prehospital providers can be trained to apply the NIHSS reliably. We hypothesize that the difference in the levels of agreement is due to the resolving deficits due to spontaneous recanalization, or worsening deficits due to evolving strokes that occur in some patients during prehospital transport. Therefore, we believe that the prehospital and hospital agreement could be higher, and can be better assessed in future studies by simultaneous application of the mNIHSS. This is likely to remove the difference in scores due to a change in clinical exam during transport to the hospital.

We describe some of the study limitations here. First, this was a pilot observational study of firefighter-paramedics within a single, urban prehospital system and so, the results are not generalizable to all prehospital systems. Second, the prehospital mNIHSS and the hospital mNIHSS were not performed at the same time and therefore, resolving or worsening neurological deficits during prehospital transport or after hospital arrival could have contributed to the differences in mNIHSS scores and the agreement. Third, we did not perform pre-test and post-post knowledge assessments for this study and therefore, we were unable to assess the immediate effect of the ENLS training on stroke knowledge. Fourth, we designed a small study since we had resource constraints and could not expand to other sites or continue this protocol. In this small study, we had a high number of large vessel occlusions and we believe that this is due to random distribution of large vessel occlusions in the population. We believe that this distribution may have normalized if we had extended the study to other ambulance agencies in San Mateo County or extended the study period. Despite these limitations, we were able to demonstrate the feasibility and the accuracy of complex stroke scale in this pilot prospective study.

## Conclusions

In summary, we were able to successfully train paramedics to use the mNIHSS in the field. Prehospital and hospital mNIHSS had a reasonable level of agreement and the prehospital mNIHSS was able to predict large vessel occlusions with moderate sensitivity. These findings provide evidence that paramedics can be trained to use complex stroke scales in the field while highlighting the need for a larger implementation study using experimental designs, to conclusively demonstrate the benefits of training and use of complex stroke scales.

## Data Availability Statement

The original contributions presented in the study are included in the article/Supplementary Material, further inquiries can be directed to the corresponding author/s.

## Ethics Statement

The studies involving human participants were reviewed and approved by Stanford University Institutional Review Board. Written informed consent for participation was not required for this study in accordance with the national legislation and the institutional requirements.

## Author Contributions

PG designed the study, obtained funding, contributed to the interpretation of the data, preparation, and approval of the manuscript. WDM, IS, JF, BL, and MC conducted the prehospital trainings, contributed to the data analysis, interpretation, manuscript review, and approval. MN, KN, and MK contributed to data analysis, interpretation, manuscript review, and approval. PG took responsibility for the entire manuscript. All authors contributed to the article and approved the submitted version.

## Conflict of Interest

The authors declare that the research was conducted in the absence of any commercial or financial relationships that could be construed as a potential conflict of interest.
