# Supplementary material for: Prehospital Identification of Large Vessel Occlusions Using Modified National Institutes of Health Stroke Scale: A Pilot Study
Source: Front Neurol. 2021 May 14;12:643356. doi: 10.3389/fneur.2021.643356 (PMC8162654; doi:10.3389/fneur.2021.643356)
Supplement: Supplementary file 1 [file Data_Sheet_1.docx]

| Patient Factors | Yes | No |
| --- | --- | --- |
| Inclusion criteria ( Include if present) |  |  |
| Age > = 18 years and |  |  |
| Acute neurological deficit consistent with suspected stroke and |  |  |
| Clinically Stable |  |  |
| Exclusion criteria (If any present, do not proceed) |  |  |
| Known decisional impairment such as dementia |  |  |
| Homeless |  |  |
| Stanford employee/student |  |  |
| Known pregnant women |  |  |
| Prisoners |  |  |
| Non English Speaking |  |  |
| Study related data/checklist for the telemedicine communication |  |  |
| Last seen normal time |  |  |
| Less than 3.5 hours |  |  |
| 3.5 hours – 7 hours |  |  |
| >7 hours |  |  |
| Blood Glucose (Normal) |  |  |
| Cincinnati Stroke Scale (Completed) –Check Yes or No |  |  |
| Face droop (Abnormal) |  |  |
| Arm drift (Abnormal) |  |  |
| Slurred speech (Abnormal) |  |  |
| Functional status of the patient at baseline: |  |  |
| Able to ambulate independently (no help from another person) w/ or w/o device |  |  |
| With assistance (person) |  |  |
| Unable to ambulate |  |  |
| Medications: Anti-platelets /Anticoagulants –Please circle |  |  |
| Aspirin, Clopidogrel (Plavix), ASA+Dipyridamole (Aggrenox),Ticagrelor, Prasugrel, Ticlopidine |  |  |
| Lovenox, Warfarin (Coumadin), Apixaban (Eliquis),argatroban, dabigatran (Pradaxa), Fondaparinux, rivoraxaban |  |  |
| Contra-indications for IV t-PA |  |  |
| Major surgery or trauma within 2 weeks |  |  |
| Known bleeding disorder, bleeding within 3 weeks |  |  |
| Acute myocardial infarction within 4 weeks |  |  |
| Previous cerebral infarction or head trauma within past three months |  |  |
