# Supplementary material for: Prehospital Identification of Large Vessel Occlusions Using Modified National Institutes of Health Stroke Scale: A Pilot Study
Source: Front Neurol. 2021 May 14;12:643356. doi: 10.3389/fneur.2021.643356 (PMC8162654; doi:10.3389/fneur.2021.643356)

**
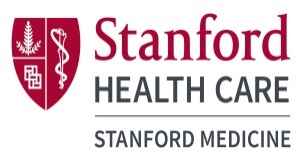
 STEP - mNIHSS Score Sheet**

| Item # | Item Name | Instructions | Scoring Guide | Patient Score |
| --- | --- | --- | --- | --- |
| 1B | LOC Questions | **“What month is it?”**  **“How old are you?”** | 0 – both correct  1 – one correct  2 – neither correct |  |
| 1C | LOC Commands | **“Make a fist and release”** (strong side)  **“Close your eyes, open them”** | 0 – both correct  1 – one correct  2 – neither correct |  |
| 2 | Gaze | **“Follow my finger”** horizontal gaze  (make sure head is not moving, hold pt’s chin)  (In a comatose patient, turn head side to side while keeping eyes open to see range of eye movements) | 0 – normal  1 – partial range (not crossing midline)  2 – total (forced deviation) to one side with no movement |  |
| 3 | Visual Fields | Tell pt **“keep looking at my nose”** and check each quadrant  **“How many fingers?”** (use 1 or 2)  (In comatose patients use “blink to threat” move hand in from the side toward the eye, look for blink) | 0 – no visual loss  1 – partial hemianopsia (one quadrant)  2 – complete hemianopsia (one side top and bottom)  3 – bilateral hemianopsia (multiple quadrants in both eyes or blind) |  |
| 11 | Neglect | 1 – (same position as testing visual fields) **“which side do you see fingers moving (R, L, both)?”**  2 – (close eyes) **“which side am I touching (R, L, both)?”** | 0 – normal  1 – mild (one modality)  2 – severe (neglect in two modalities or ignoring one side) |  |
| 8 | Sensory | Ask pt to open eyes, light touch to arms  **“Do you feel this (touch R)? Do you feel this (touch L)? Does it feel the same on both sides?”** | 0 – normal  1 – abnormal |  |
| 5a | Left Arm Motor | Hold arm at 45 degrees for **10 seconds**, palm down  Encourage patient if starting to drift or if poor effort | 0 – no drift  1 – drift but doesn’t hit bed  2 – hits bed  3 – no effort against gravity (moves in lateral plane)  4 – no movement |  |
| 5b | Right Arm Motor | Same as 5a (Use R arm)  Encourage patient if starting to drift or if poor effort | 0 – no drift  1 – drift but doesn’t hit bed  2 – hits bed  3 – no effort against gravity (moves in lateral plane)  4 – no movement |  |
| 6a | Left Leg Motor | Hold leg at 45 degrees above bed for **5 seconds**  Encourage patient if starting to drift or if poor effort | 0 – no drift  1 – drift but doesn’t hit bed  2 – hits bed  3 – no effort against gravity (moves in lateral plane)  4 – no movement |  |
| 6b | Right Leg Motor | Same as 6a, use R leg  Encourage patient if starting to drift or if poor effort | 0 – no drift  1 – drift but doesn’t hit bed  2 – hits bed  3 – no effort against gravity (moves in lateral plane)  4 – no movement |  |
| 9 | Language | Use stroke card  Name the objects and describe the situation in the picture | 0 – normal  1 – mild aphasia/able to maintain a simple conversation  2 – severe aphasia/ broken sentences  3 – mute or global aphasia |  |
|  |  | mNIHSS=> 5, consider LVO alert | TOTAL SCORE |  |


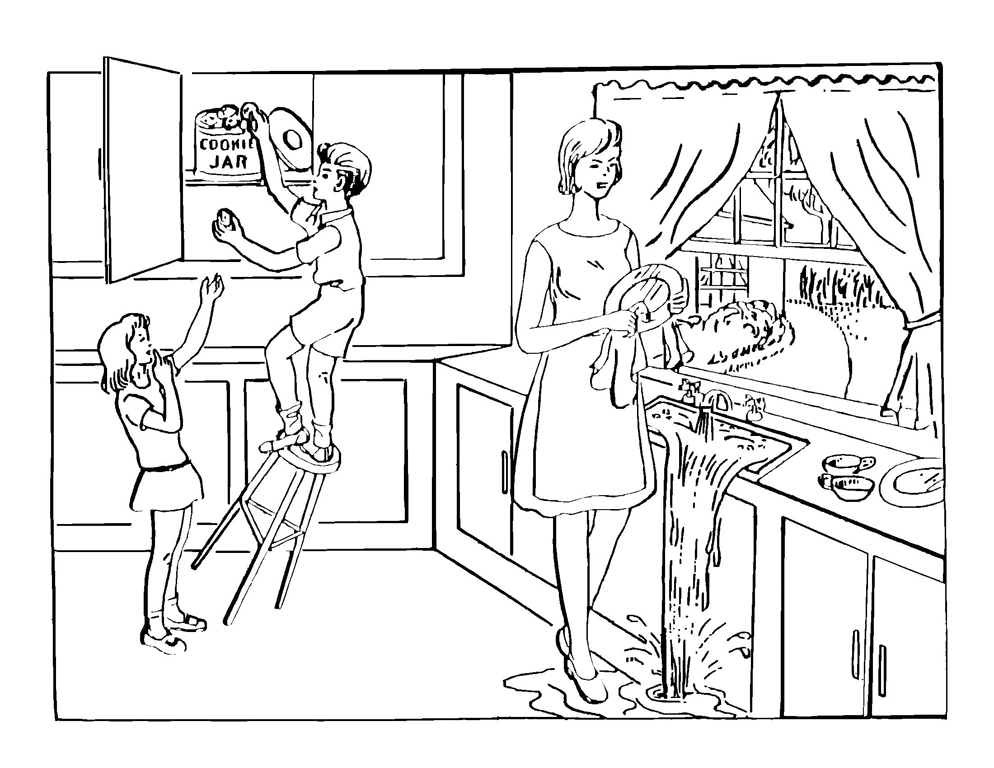


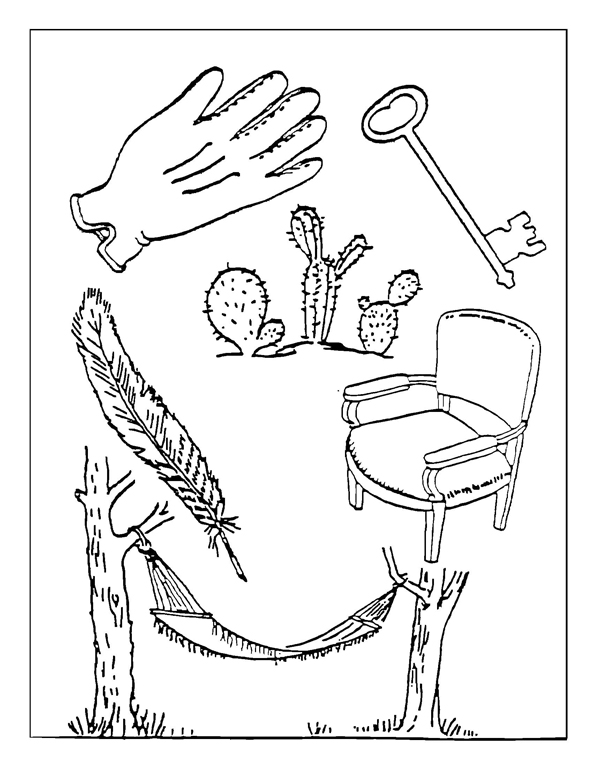

Supplement: Supplementary file 2 [file Data_Sheet_2.DOCX]
